# Novel Drug Delivery Systems for Phytomedicines

**DOI:** 10.3390/pharmaceutics16081059

**Published:** 2024-08-12

**Authors:** Xingwang Zhang, Wei Wu

**Affiliations:** 1 Department of Pharmaceutics, School of Pharmacy, Jinan University, No. 855 East Xingye Avenue, Guangzhou 511443, China; 2 Center for Medical Research and Innovation, Shanghai Pudong Hospital, Fudan University Pudong Medical Centre, Shanghai 201399, China; 3 Key Laboratory of Smart Drug Delivery, Ministry of Education, School of Pharmacy, Fudan University, Shanghai 201203, China

Many active pharmaceutical ingredients (APIs) are found in or originate from phytomedicines. Traditional Chinese medicine has loomed large for thousands of years and still stands in the world of medicine. Notably, herbal and botanical medicines also face serious formulation challenges due to deficiencies in their own physicochemical properties and the occurrence of adverse reactions. Novel drug delivery systems dramatically contribute to drug delivery efficiency and bioaccessibility [1], and a variety of phytomedicines have shown excellent therapeutic potential with the help of novel drug delivery systems. Novel drug delivery systems, including but not limited to lipid-based nanocarriers, micelles, micro/nanoemulsions, polymeric nanoparticles, polymer–lipid hybrid nanoparticles, and organometallic nanocarriers, enable the efficient delivery of intractable drugs to the target site, resulting in effective therapeutic concentrations [2,3]. To this end, this Special Issue invites eminent experts and scholars from home and abroad to contribute their original research articles and reviews on this multifaceted realm.

In this Special Issue collection (https://www.mdpi.com/journal/pharmaceutics/special_issues/S0Z47J7Y1W, accessed on 30 April 2024), a total of six articles were contributed, including three reviews and three research papers, and readers will be informed about the latest advancements in phytomedicine delivery. Topics related to this topic include the development of phytocomponent-modified hydrogel coatings, phytomedicine delivery via selenized polymer–lipid hybrid nanoparticles, the application of spanlastics (elastic vesicular drug carrier systems), advances in oleanolic acid drug delivery, transdermal delivery strategies involving colchicine, and the mechanism by which nanoparticles cross the intestinal epithelium.

Modified hydrogel interfaces are promising formats for cell culture because they can mimic the extracellular matrix (ECM), which guides cellular functions and influences cell behavior. Hydrogels offer an aqueous microenvironment akin to the ECM, incorporating essential features such as cell adhesion sites and promoting growth factor diffusion. Xu et al. [4] prepared tannic acid-modified poly(*N*-isopropylacrylamide) hydrogel coatings in situ to increase the cell response. Tannic acid (TA) is a naturally occurring polyphenol that can regulate the behaviors of various bioactive factors involved in cell culture, and the incorporation of this compound into PNIPAAm coatings may enhance protein interactions and increase cell adhesion without requiring precise control of the coating thickness. The customized PNIPAAm860-APTES-TA and PNIPAAm500-APTES-TA enhanced protein adsorption, extended protein retention, and maintained protein viability by inhibiting TA release. As a result, the adhesion, viability, and proliferation of the cells grown on these surfaces were improved, and owing to their interfacial superhydrophilicity, TA-modified hydrogel coatings exhibit enhanced efficiency in cell culture applications.

The oral administration of insoluble and enterotoxic drugs is often hindered by gastrointestinal irritation, side effects, and low bioavailability. Tripterine (Tri) is a leading phytomedicine in anti-inflammatory research, although it suffers from poor water solubility and biocompatibility. To increase its effectiveness against enteritis, Ren et al. [5] fabricated selenized polymer–lipid hybrid nanoparticles for optimized oral delivery, and compared with unmodified Tri-PLNs, Se@Tri-PLNs resulted in a slower drug release and greater stability in digestive fluids, with increased cellular uptake in Caco-2 cells, as confirmed via flow cytometry and confocal microscopy. The oral bioavailability of Se@Tri-PLNs was up to 397% relative to Tri suspensions; furthermore, Se@Tri-PLNs exhibited more significant in vivo anti-enteritis effects, leading to a substantial resolution of ulcerative colitis. Polymer–lipid hybrid nanoparticles (PLNs) create drug supersaturation in the gut and sustain Tri release, while selenium surface engineering enhances formulation performance and anti-inflammatory efficacy. This work provides a proof-of-concept for a combined therapy for inflammatory bowel disease (IBD) via the use of phytomedicine and selenium in an integrated nanosystem.

Spanlastics are a novel type of elastic vesicular nanocarrier made from surfactants, consisting of spans and edge activators. To enhance the nose-to-brain delivery of piperine (PIP), Gupta and colleagues formulated this phytomedicine into Spanlastics on the basis of the Box–Behnken design (BBD) [6]. Compared with the PIP suspensions, the developed formulation enhanced the permeation of PIP across the nasal mucosa. PIP-SPL achieved a higher *C*_max_ and *AUC* via the intranasal route than via oral administration. Compared with standard diazepam, PIP-SPL has significant antiepileptic potential. Moreover, the PIP-SPL formulation was safe for intranasal use. The study thus supports the conclusion that the resulting spanlastic vesicles represent an effective carrier for PIP intranasal delivery in epilepsy management.

The increasing interest in oleanolic acid (OA), a triterpenoid with notable health benefits, highlights the need for its efficient application in the clinic. OA has diverse pharmacological activities, including antidiabetic, anti-inflammatory, immune-enhancing, gastroprotective, hepatoprotective, antitumor, and antiviral properties. Nevertheless, to unlock its therapeutic potential, significant challenges must be addressed. Wasim and Bergonzi contributed a review article to elaborate the advancements in delivery systems for OA [7]. Novel formulation strategies, such as lipid-based delivery systems (SLNs, NLCs, micro- and nanoemulsions, and liposomes), micelles, nanosuspensions, nanoparticles, solid dispersions, phospholipid complexes, and others, have been introduced.

Colchicine (COL) is a natural drug known for its strong anti-inflammatory effects. However, its narrow therapeutic index limits its clinical use due to serious gastrointestinal side effects, with only oral formulations currently available globally. Gao’s group identified novel transdermal, injectable, and oral delivery methods as promising formulations for COL administration [8]. They discussed advancements in these delivery strategies focusing on synergy and attenuation, highlighting that transdermal delivery can bypass the first-pass effect and avoid the pain associated with oral and injection routes. Therefore, this approach shows significant promise, warranting further research into effective COL delivery formulations.

NPs play a crucial role in the oral delivery of phytomedicines, making it essential to understand how they traverse the intestinal epithelium for the rational design of nanomedicines. Through a literature review, Tu led his team to elucidate the transepithelial transport mechanism of nanoparticles [9]. The ability of nanoparticles to cross the intestinal epithelial membrane strongly influences their post-enterocyte absorption ability. Tu et al.’s review discussed the behavior of nanoparticles in the intestinal epithelial cell membrane, emphasizing their intracellular mechanisms, and three key processes are outlined: uptake by epithelial cells at the apical side, intracellular transport, and exocytosis at the basal side. The work by Tu and his team will enable practitioners to understand the in vivo behavior of nanoparticles in the intestinal epithelial cell membrane, aiding in the development of novel phytomedicine formulations.

Today, herbal remedies are on the rise, and this Special Issue collection illustrates how challenges in delivering phytomedicines are fostering the ongoing innovation in formulations. Advanced formulation strategies and new biomaterial applications provide new insight into the potentiation of phytomedicines, and the contributions to this Special Issue not only mirror the latest developments in phytomedicine delivery but also underscore the importance of interdisciplinary research. Despite the numerous issues associated with the delivery of phytomedicines, novel drug delivery systems are consistently providing unexpected solutions.
